# A randomized controlled trial of daclizumab *versus* anti-thymocyte globulin induction for heart transplantation

**DOI:** 10.1186/2047-1440-3-14

**Published:** 2014-07-30

**Authors:** John C Mullen, Emily J Kuurstra, Antigone Oreopoulos, Michael J Bentley, Shaohua Wang

**Affiliations:** 1 Division of Cardiac Surgery, University of Alberta Hospital, Edmonton, AB, Canada; 2 Division of Cardiac Surgery, University of Alberta Hospital, 2D2.18 WMC, 8440 112 Street, Edmonton, AB T6G 2B7, Canada

**Keywords:** Heart transplantation, Induction therapy, Immunosuppression, Daclizumab, Anti-thymocyte globulin

## Abstract

**Background:**

The purpose of this study was to test the efficacy and safety of daclizumab (DZM) *versus* anti-thymocyte globulin (ATG) as a component of induction therapy in heart transplant recipients.

**Methods:**

Thirty heart transplant patients were randomized to receive either ATG or DZM during induction therapy. Patients in the DZM group received an initial dose of 2 mg/kg intravenous (IV) at the time of transplant and 1 mg/kg IV on postoperative day 4.

**Discussion:**

Recipient, donor, and intraoperative variables did not differ significantly between groups. The cost of induction therapy, total drug cost, and hospital ward costs were significantly less for the DZM group. Average absolute lymphocyte and platelet counts were significantly higher in the DZM group. There were no significant differences in the incidence of rejection, infection, malignancy, or steroid-induced diabetes. One year survival was excellent in both groups (87%, *P* = 0.1). Daclizumab is a safe component of induction therapy in heart transplantation.

## Background

Cardiac transplantation remains a definitive treatment option for patients with end-stage heart disease. Survival rates have improved dramatically. Nonetheless, progress in immunosuppression has been slower, partly because the heart is a fundamental organ and acute allograft rejection can include hemodynamic compromise, irreversible graft injury, and death. Furthermore, the immunosuppressive therapy used to prevent rejection increases the risk of infection, which continues to be a leading cause of death in the first year after cardiac transplantation [1,2]. A common immunosuppression protocol for cardiac transplantation includes cyclosporine, mycophenolate mofetil, and corticosteroids (triple therapy). An alternative to standard triple therapy at the time of cardiac transplantation has been the use of augmented immunosuppression, commonly termed ‘induction therapy’. Induction agents consist of antibodies that exhibit protective effects from allograft rejection; they are administered during the immediate postoperative period when the risk of rejection is highest due to a high donor leukocyte load [3]. Data from the International Society of Heart and Lung Transplant (ISHLT) show that 47% of adult heart transplant patients in the first 6 months of 2012 received some type of induction therapy [1]. Either a polyclonal anti-lymphocyte/anti-thymocyte globulin or an interleukin-2 (IL-2) receptor antagonist was utilized in most protocols; however, the type of product used, its dosage, and the duration of administration varied greatly. At present, there is no general consensus on the best method of induction. This fact has prompted the development of new immunosuppressive agents designed to reduce the incidence of acute rejection.

Daclizumab (DZM) is a novel compound for use as a component of induction therapy. This agent is a murine monoclonal antibody, directed at the alpha subunit of the interleukin-2 receptor (IL-2R) expressed on activated T-lymphocytes [4]. Ninety percent of the murine protein structures have been replaced with human amino acid sequences through genetic engineering. It therefore does not induce a clinically relevant response by the host immune system. DZM was approved by Health Canada and the Federal Drug Administration (FDA) for prophylactic use of acute organ rejection in patients receiving renal transplants. Our induction therapy included T-lymphocyte inactivation through the administration of polyclonal anti-thymocyte globulin (ATG). There have been no reported randomized controlled trials comparing DZM to ATG induction in heart transplantation. The purpose of this study was to compare these therapies in heart transplant recipients.

## Methods

All adults listed for heart transplantation between June 2001 and April 2005 were considered for the study. Exclusion criteria included emergent surgery, previous transplant, multiple-organ transplant including heart-lung transplant, active infection, hepatitis C, high positive panel reactive antibodies (>15%), known sensitivity to DZM, ATG, or mouse antigens, expected inability to be followed at the study center for a full year, and inability to give informed consent. Ethical approval was obtained from the University of Alberta Health Research Ethics Board.

A total of 30 adult heart transplant recipients were randomized to receive either DZM (Hoffman-La Roche Ltd., ON, Canada) or ATG (Pharmacia & Upjohn Inc., ON, Canada) as part of induction therapy. Randomization was generated by computer. Enrolment and assessment of outcomes were performed by two research assistants. Only patients were blinded to the treatment. The primary endpoints of this study were the number and severity of infection episodes post-transplant. Secondary endpoints included incidence of rejection, survival, and cost.

### Immunosuppressive regimen

Patients in the control group received 10 mg/kg intravenous (IV) ATG beginning postoperatively and infused continuously for 5 to 7 days until cyclosporine or tacrolimus reached therapeutic levels. Patients in the treatment group received DZM IV at 2 mg/kg within 4 h postoperatively followed by a single 1 mg/kg dose on postoperative day 4. Patients in both groups received methylprednisolone (Solu-Medrol®, Novopharm, ON, Canada) 1 g IV intraoperatively, followed postoperatively by 2 mg/kg IV every 12 h for three doses. This was followed by prednisone or methylprednisolone (depending on whether the patient could tolerate oral medication) 1 mg/kg daily. This was tapered by 2 mg/day to 0.3 mg/kg/day. Mycophenolate mofetil (CellCept®, Hoffman La-Roche, ON, Canada) was given preoperatively 1,000 mg per oral or IV followed by 1,000 mg IV twice daily postoperatively until the patient could tolerate oral medication. At this time the patient was switched to mycophenolate mofetil 1,000 mg per oral twice daily, with a target dose of 3 g daily. Patients treated with cyclosporine received cyclosporin A (Neoral®, Novartis Pharmaceuticals Canada Inc., QB, Canada) 150 mg to 300 mg per oral twice daily until therapeutic levels were reached (250 μg/L to 400 μg/L). Patients treated with tacrolimus (Prograf®, Astellas Pharma Canada, Inc., ON, Canada) received tacrolimus 2 mg to 5 mg per oral twice daily until therapeutic levels were reached (10 mg/mL to 15 mg/mL). Patients in the ATG group received a pulse of methylprednisolone 2 mg/kg IV every 12 h for three doses starting at the point of ATG discontinuation.

### Infection prophylaxis

Patients with Epstein-Barr virus (EBV) or cytomegalovirus (CMV) donor-seropositive/recipient-seronegative received 900 mg each day for 14 weeks of oral ganciclovir (Cytovene®, Hoffman-La Roche Ltd., ON, Canada) or valgancyclovir (Valcyte®, Hoffman-La Roche Ltd, ON, Canada) therapy. Patients who were CMV donor seropositive/recipient seropositive or donor seronegative/recipient seropositive received 2 weeks of 900 mg twice per day of oral ganciclovir or valganciclovir therapy.

### Diagnosis and treatment of acute and chronic rejection

Acute rejection was defined as either biopsy-proven as defined by ISHLT grade 3R (3A or 3B) or higher histology [5], suspected and subsequently treated rejection in the presence of hemodynamic compromise, or grade 1A or 1B with symptoms (reduced ejection fraction, shortness of breath, decreased voltages or a gallop rhythm). Treatment of acute rejection typically consisted of intravenous methylprednisolone 500 g to 1,000 g for 3 days. Severe high grade or humoral rejection was treated with plasmaphoresis, intravenous immune globulin, ATG, or RATGAM (ATG made from rabbits). Grade 2 rejection or symptomatic low grade (1A or 1B) rejection was treated with a 50 mg to 80 mg prednisone tapering dose. Heart transplant patients at our centre receive 13 biopsies during the first year post transplant.

### Diagnosis of infection

Infection was considered significant if it resulted in symptoms and/or a change in medical management. An infection was also considered to be severe if it appeared to prolong hospitalization, required re-admission to hospital, or was treated with intravenous antibiotics after initial hospitalization.

### Cost analysis

Cost data were determined by calculating total drug cost, ICU cost, and ward cost. Drug costs were obtained directly from the pharmacy department. ICU and ward costs were based on a study by Hamilton et al. [6], in which hospital costs were acquired from patient resource consumption profiles. This accounting method was developed at our center. It included nursing costs, the direct and indirect labor and supply costs related to nursing, laboratory, radiological, and rehabilitative medicine costs, and all direct and indirect labor and supply costs required to perform tests or procedures. Physician fees were not included.

### Statistics

Statistical analysis was performed using SPSS software (SPSS Inc., Chicago, IL, USA). All analysis was based upon an intention to treat principle. Continuous variables were compared between groups by an independent *t*-test or Mann-Whitney U where non-parametric analysis was appropriate. Discrete variables were compared between groups using chi-squared and Fisher’s exact tests where appropriate. Survival curves were created with the Kaplan-Meier method with log-rank comparisons between groups. Results of continuous variables are presented as mean ± standard error. The alpha level was set at *P* ≤0.05. A study by Sarris et al. [7] revealed a 73% 1-year infection rate in heart transplant recipients. A sample size of 14 patients per group was determined to detect a 43% reduction in infection rate with an alpha error of 5% and a power of 80%.

## Results

**Figure 1:**
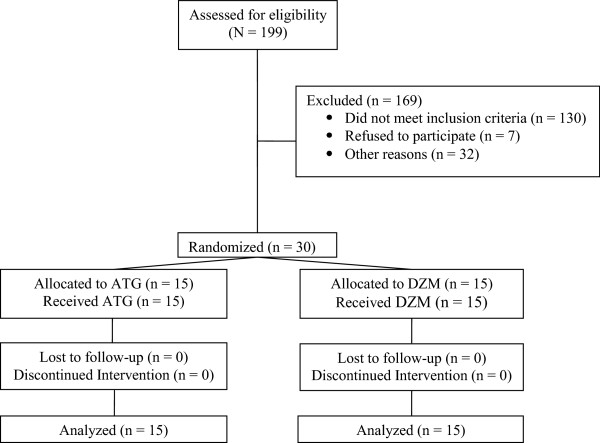
CONSORT diagram.

The flow of participants through the study is presented in Figure 1. One hundred and ninety-nine patients were assessed for eligibility: 130 were deemed ineligible due to exclusion criteria, seven declined, 32 did not participate because they did not receive a transplant during the study period, and the remaining 30 were randomized. There were no drop-outs.

A summary of recipient demographics and perioperative outcomes are presented in Table 1. There were no significant differences in preoperative recipient demographics. The incidence of cytomegalovirus (CMV) and Epstein-Barr virus (EBV) mismatch was similar between groups. Patients in the DZM group tended to require more inotropic support postoperatively (higher inotropic severity score: DZM 65 ± 5, ATG 49 ± 6, *P* = 0.07). No other statistically significant differences were observed in intraoperative and immediate postoperative outcomes. There were also no significant differences in donor demographics between groups (Table 2).

**Table 1:** Recipient demographics and perioperative outcomes

	**ATG (*n*** **= 15)**	**DZM (*n*** **= 15)**	***P *value**
Age (years)	58 ± 3	57 ± 3	0.9
Sex (male/female)	11/4	12/3	1.0
Diagnosis			
Idiopathic cardiomyopathy	11 (73%)	10 (67%)	1.0
Other	4 (27%)	5 (33%)	1.0
Height (cm)	172 ± 2	172 ± 3	0.9
Weight (kg)	78 ± 3	83 ± 5	0.4
Body mass index (kg/m^2^)	26 ± 1	28 ± 2	0.4
Status			
1: Stable and waiting out of hospital	8 (53%)	9 (60%)	1.0
2: Stable and waiting in hospital	3 (20%)	2 (13%)	1.0
3: In hospital on Inotropic support	4 (27%)	3 (20%)	1.0
4: Intubated	0 (0%)	1 (7%)	1.0
Diabetes mellitus	0 (0%)	2 (13%)	0.5
Lymphocytotoxic crossmatch			
Negative	15 (100%)	15 (100%)	1.0
CMV mismatch			
Negative recipient/Positive donor	1 (7%)	1 (7%)	1.0
EBV mismatch			
Negative recipient/Positive donor	0 (0%)	0 (0%)	-
Operative time (min)	333 ± 18	351 ± 20	0.5
Cardiopulmonary bypass time (min)	187 ± 10	194 ± 16	0.7
Intubation time (h)	96 ± 47	130 ± 55	0.7
Intensive care unit time (h)	264 ± 102	289 ± 96	0.9
Inotropic severity score	49 ± 6	65 ± 5	0.07
Total hospital length of stay (days)	29 ± 8	26 ± 6	0.8

CMV: cytomegalovirus; EBV: Epstein-Barr virus.

**Table 2:** Donor characteristics

	**ATG (*n*** **= 15)**	**DZM (*n*** **= 15)**	***P *value**
Age (years)	35 ± 5	35 ± 4	0.9
Sex (male/female)	11/4	10/5	1.0
Height (cm)	172 ± 3	172 ± 3	0.9
Weight (kg)	78 ± 5	86 ± 4	0.2
Body mass index (kg/m^2^)	26 ± 1	29 ± 1	0.1
Donor/recipient weight ratio	1.01 ± 0.05	1.08 ± 0.09	0.5
Donor ischemic time (min)	254 ± 22	249 ± 24	0.9

Postoperative laboratory and drug administration values averaged over a 10-day post-transplant period are presented in Table 3. Average absolute lymphocyte counts were significantly higher in the DZM group (0.89 × 10^9^/L *vs.* 0.45 × 10^9^/L, *P* <0.0001), as well as average platelet count (153 per mm^3^*vs.* 114 per mm^3^, *P* = 0.004). In addition, average chloride was higher in the DZM group (103 ± 1 mmol/L *vs.* 101 ± 1 mmol/L, *P* = 0.05). In the control group, ATG was infused for 7 ± 2 days. As expected, volume of ATG given intravenously was significantly higher than DZM (5,934 ± 669 mL *vs.* 942 ± 152 mL, *P* <0.0001), and methylprednisolone dose was significantly less in the DZM group (495 ± 38 mg *vs.* 1,242 ± 278 mg, *P* <0.0001). Other drug dosages and volumes were similar between groups.

**Table 3:** Postoperative laboratory data and drug administration

	**ATG (n = 15)**	**DZM (n = 15)**	**p value**
Average white blood cells (×10^9^/L)	16.6 ± 1.3	16.4 ± 1.2	0.9
Average neutrophils (×10^9^/L)	13.9 ± 1.0	13.8 ± 0.9	0.9
Average absolute lymphocytes (×10^9^/L)	0.45 ± 0.04	0.89 ± 0.09	<0.0001
Average red blood cells (×10^9^/L)	3.3 ± 0.1	3.2 ± 0.1	0.2
Average platelet count (per mm^3^)	114 ± 9	153 ± 8	0.004
Average hemoglobin (g/L)	10.3 ± 0.2	9.9 ± 0.2	0.2
Average sodium (mmol/L)	136 ± 1	137 ± 1	0.6
Average potassium (mmol/L)	4.2 ± 0.1	4.2 ± 0.1	0.7
Average chloride (mmol/L)	101 ± 1	103 ± 1	0.05
Average CO_2_ (mmol/L)	25 ± 1	24 ± 1	0.2
Average glucose (mmol/L)	8.4 ± 0.4	8.7 ± 0.7	0.6
Average urea (mmol/L)	16.2 ± 1.3	16.8 ± 1.2	0.7
Average ionized calcium (mmol/L)	1.25 ± 0.21	1.16 ± 0.02	0.09
Average creatinine (mmol/L)	143 ± 10	178 ± 21	0.1
Platelet units given	8 ± 2	7 ± 4	0.9
Red blood cell units given	9 ± 2	8 ± 3	0.8
Study drug induction volume (mL)	5,934 ± 669	942 ± 152	<0.0001
Methylprednisolone (mg)	1,242 ± 278	495 ± 38	<0.0001
Prednisone (mg)	552 ± 38	626 ± 53	0.2
IV Mycophenolate Mofetil (mg)	6,017 ± 788	6,000 ± 1005	1.0
p.o. Mycophenolate Mofetil (mg)	14,983 ± 1025	16,317 ± 1145	0.4
Patients receiving cyclosporin A only	12	11	1.0
Patients receiving tacrolimus only	2	1	1.0
Patients converted from cyclosporin A to tacrolimus	0	3	0.2
Patients converted from tacrolimus to cyclosporin A	1	0	1.0
Cyclosporin A (mg)	2,532 ± 348	2,621 ± 333	0.8
Tacrolimus (mg)	40 ± 14	40 ± 6	1.0
Insulin (units)	502 ± 80	724 ± 202	0.3
Total steroids for 1 year (mg)	4,631 ± 638	3,846 ± 434	0.2

**Figure 2:**
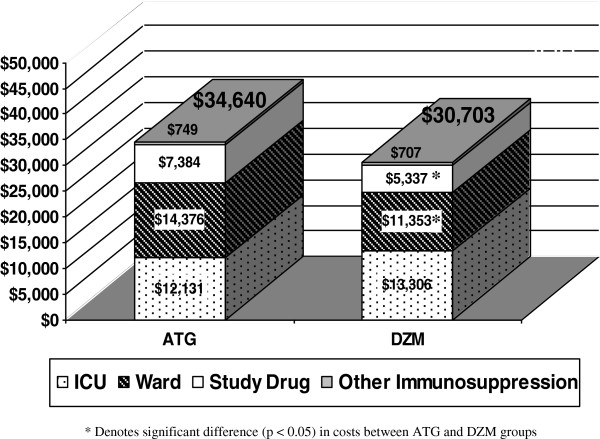
**Cost analysis: total hospital cost (*P*** **= 0.8).**

The cost analysis is illustrated in Figure 2. Induction cost (cost of DZM *vs.* cost of ATG) was significantly lower in the DZM group (Figure 2, $5,337 ± 308, CI ± 604.17 *vs.* $7,384 ± 799, CI ± 1,565.84, *P* = 0.03). Total drug cost (induction cost plus methylprednisolone, mycophenalate mofetil, cyclosporine A and/or tacrolimus, and prednisone) was also significantly lower in the DZM group (Figure 2, $6,044 ± 328, CI ± 642.28 *vs.* $8,133 ± 828, CI ± 1,622.97, *P* = 0.03). In addition, hospital ward (step-down unit) cost was lower in the DZM group (Figure 2, $11,353 ± 3,320, CI ± 6,507.38 *vs*. $14,376 ± 3,526, CI ± 6,911.53, *P* <0.05). Intensive care unit stay and total hospital costs were not significantly different between groups (Figure 2).

The incidence of rejection is presented in Table 4. Mean biopsy grade was lower in the DZM group, but not statistically different (0.3 *vs.* 0.4, *P* = 0.09). Allograft rejection occurred in two patients, both in the ATG group. One of these patients had confirmed humoral rejection 19 days post transplant, and was treated with IV immune globulin. The second patient experienced an episode of hypotension 6 days post transplant with right ventricular dysfunction and right bundle branch block with decreased voltages. This was felt to be due to acute rejection, and the patient was subsequently treated with pentaspan, inotropes, IV cyclosporine A, and pulse steroids.

**Table 4:** Rejection, infection, and other outcomes

	**ATG (n = 15)**	**DZM (n = 15)**	**p value**
Mean biopsy grade	0.4	0.3	0.09
Patients experiencing rejection	2 (13%)	0	0.5
Total number of acute rejections	2	0	0.2
Time to first rejection episode (days)	84	-	-
Patients experiencing infection	10 (67%)	10 (67%)	1.0
Total number of infections	25	21	0.7
Infections/patient	1.7	1.4	0.7
Patients experiencing severe infection	4 (27%)	5 (33%)	1.0
Number of severe infections	7	7	1.0
Severe infections/patient	0.5	0.5	1.0
Number of CMV infections	1	2	1.0
Malignancy	0	1	1.0
Steroid-induced diabetes	2	2	1.0
Re-transplant	0	0	-
ICU length of stay (days)	11 ± 4	12 ± 4	09
Total hospital length of stay (days)	29 ± 8	27 ± 6	0.8
One-month survival	93%	100%	0.1
One-year survival	87%	87%	0.1

The number of patients experiencing at least one episode of infection was the same between groups (Table 4, 67% in both groups). Time to first infection and other infectious complications were also similar between the two groups.

**Figure 3:**
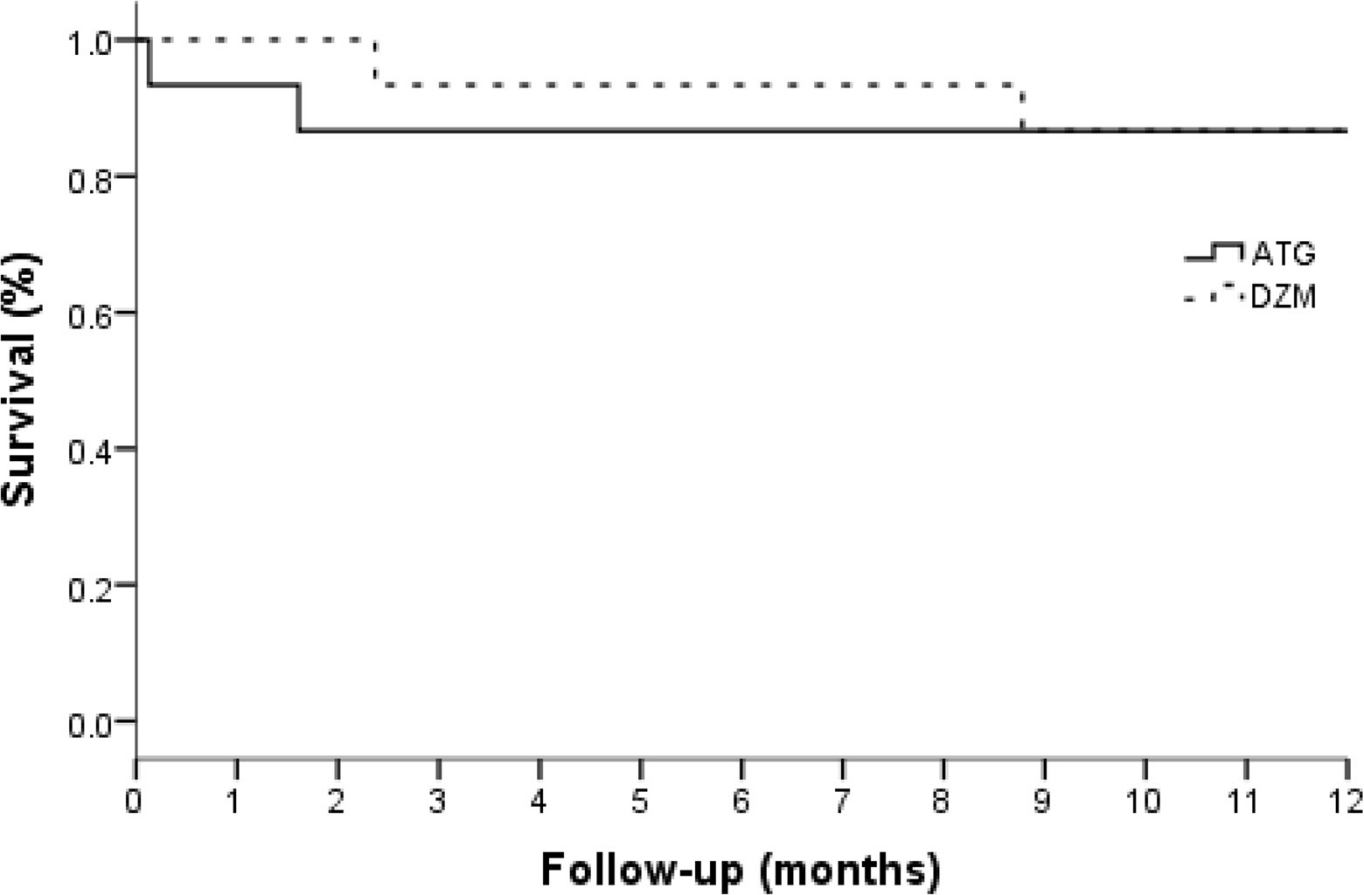
**Actuarial survival.** Log rank comparison, *P* = 0.1.

No patient had any acute side effect or allergic reaction to either study drug. There was no significant difference for incidence of steroid induced-diabetes. None of the study patients were re-transplanted. One of the patients in the DZM group had an incidence of malignancy: a basal cell carcinoma lesion on the ear which was treated successfully.An actuarial survival curve is presented in Figure 3. Survival at 1 month and 1 year was 100% and 87% in the DZM group, and 93% and 87% in the ATG group, respectively. There were two patients who died in the ATG group. The first patient in the ATG group died 5 days post transplant due to intestinal ischemia. The second patient in the ATG group died 49 days post transplant due to fungal sepsis. Two patients also died in the DZM group. The first patient died 72 days post transplant due to sepsis. The second patient in the DZM group died 267 days post transplant of a stroke.

## Discussion

Infection and rejection have been identified as risk factors for morbidity and mortality after heart transplantation [1]. In order to improve patient survival and quality of life, strategies have been developed to minimize these risk factors for infection and rejection, including induction agents as part of the immunosuppression regimen in the early postoperative period. This study compared the results of using DZM *versus* ATG during induction therapy after heart transplantation.

The use of DZM in addition to a triple immunosuppressive regimen was well tolerated in heart transplant recipients, with one adverse reaction to the drug. There were no differences in the incidence of rejection, steroid-induced diabetes or malignancy compared to patients who received ATG. In addition, average absolute lymphocytes and average platelet count were significantly higher in the DZM group. One-year survival was excellent in both groups (87%) and was similar to the experience from the ISHLT Data Registry (1-year survival 81% based on survival rates for heart transplants performed between 1982 and 2011 [1].

The efficacy and safety of DZM has been demonstrated in a large number of kidney [8-28], kidney-pancreas [29,30], liver [31-36], and lung clinical trials [37,38]. There have been few studies involving DZM in cardiac transplantation [39-45], despite the observation that almost 50% of patients undergoing cardiac transplantation receive anti-body-based induction therapy [1].

In our previous study of ATG and DZM in lung transplant recipients [37], both agents were also equally effective in rejection outcomes, however, the time to first rejection tended to be more prolonged with DZM (ATG: 138 days, DZM: 220 days, *P* = 0.06).

The incidence of overall infection in the present study is similar to other reports in heart transplantation [7]. DZM has not been found to alter infection rates in kidney [9,12,15,21,23,46], kidney-pancreas [28-30], heart [42,45], lung [37,38,47,48] or liver [31,34,35,49,50] transplant recipients.

The results of this study support the efficacy of a two dose DZM regimen which is simpler in that patients need not return to hospital for treatment every 2 weeks. The ATG regimen is more complex than our DZM regimen, requiring 5 to 7 days of continuous intravenous infusion and more steroid administration. In addition, ATG may have limited use due to the formation of antibodies; therefore, treatment of future rejection episodes may not be possible with ATG.

In this study, both average absolute lymphocyte count and platelet count were significantly reduced in the ATG group compared to the DZM group (Table 3). This finding is consistent with our previous study of the two agents in lung transplant recipients [37]. Brock and colleagues [38] noted that in lung transplantation, patients receiving ATG induction most commonly develop thrombocytopenia, with 74% developing a platelet count of <100,000/mm^3^[38]. In our current study, one patient in the ATG group developed severe thrombocytopenia, however, not in response to the ATG infusion.

The exact mechanism of effect of DZM is unknown; however, the efficacy of DZM is likely related to its selective targeting of active T-lymphocytes. DZM readily binds to the alpha subunit of the IL-2 receptor of circulating active T-lymphocytes, preventing activation of inactive T-lymphocytes by stimulation of the IL-2 receptor and possibly causing down regulation of IL-2 receptor expression [51,52]. This allows DZM to specifically target the active lymphocytes, leaving the immune system otherwise intact. This is consistent with our results of higher average absolute lymphocytes in the DZM group. DZM has also been genetically engineered to contain 90% human determinants. This reduces the immunogenicity of the molecule and lengthens its circulating half-life (20 days). An advantage of DZM’s long half-life is that T-cell rebound after discontinuation of DZM does not occur. Patients receiving ATG at our center receive a pulse of methylprednisolone at the point of ATG discontinuation to prevent this T-cell rebound. Patients in the ATG group therefore required a significantly higher dose of methylprednisolone compared to the DZM group. Furthermore, because only a fraction of the antibodies from ATG are directed against T-lymphocytes, a large amount of volume (10 mg/kg for 5 to 7 days) must be administered. This extra volume may lead to excess fluid balances which we normally try to avoid after heart transplantation.

A cost analysis revealed that the cost of DZM induction was significantly lower than ATG induction in heart transplant recipients. Total drug cost and hospital ward cost was also less in the DZM group. The use of DZM induction could thus lead to a cost savings of between $2,000 and $3,000 in some heart transplant recipients.

Our study has demonstrated that DZM was a safe component of induction therapy in heart transplantation. Our study highlights the advantages of DZM, including ease of administration, lower cost, higher lymphocyte count, and freedom from excessive platelet destruction. Both methods of induction therapy worked well with excellent 1-year survival. Daclizumab was a useful induction agent in our immunosuppression protocol for heart transplant recipients.

## Competing interests

This study was funded by an unrestricted research grant from Hoffmann-La Roche. Data collection, analysis, and manuscript preparation was conducted by the investigators in compliance with the protocol and was independent of the sponsor. The authors declare that they have no competing interests.

## Authors’ contributions

JCM, AO, and MJB participated in research design. JCM, AO, MJB, and SW participated in acquisition of data. EJK, AO, and MJB, participated in data analysis. JCM, EJK, AO, and MJB participated in writing of the manuscript. All authors read and approved the final manuscript.
